# Supplementary material for: The Anti-Inflammatory and Skin Barrier Function Recovery Effects of Schisandra chinensis in Mice with Atopic Dermatitis
Source: Medicina (Kaunas). 2023 Jul 24;59(7):1353. doi: 10.3390/medicina59071353 (PMC10385087; doi:10.3390/medicina59071353)
Supplement: Supplementary file 1 [file medicina-59-01353-s001.zip › medicina-2499289-supplementary.pdf]

Supplementary Materials: The Anti-Inflammatory and Skin Barrier Function Recovery Effects of *Schisandra chinensis* in Mice with Atopic Dermatitis

Mice were randomly divided into 6 groups and shaved on day 1. MC903 (4 nmol/day) was applied onto the shaved dorsum of mice for 8 days (days 4-11). EESC (60, 180, or 600 μg/day) and DEX (150 μg/day) were applied topically for 6 consecutive days (days 6-11). All animals were sacrificed on day 12. The experimental process is summarized in supplementary data (supplementary data 1.)

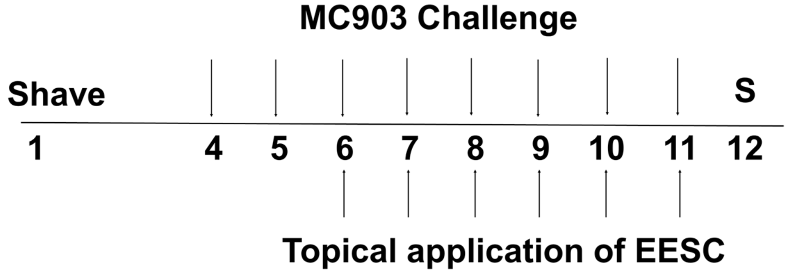

Figure S1. Experimental schedule. EESC, ethanol extract of *Schisandra chinensis*

Table S1. Target primers used for qPCR in this study

Target gene	Primer sequences (5′ to 3′ direction)
IL-1β	Forward: CAGGCAGTATCACTCATTGT<br>Reverse: CCAGCAGGTTATCATCATCA
IL-4	Forward: GAGAGAGATCATCGGCATTT<br>Reverse: GCTCCATGAGAACACTAGAG
IL-6	Forward: GCCAGAGTCCTTCAGAGAGA<br>Reverse: GGTCTTGGTCCTTAGCCACT
IL-8	Forward: CATCCAGAGCTTGAGTGTGA<br>Reverse: GTTAGCCTTGCCTTTGTTCA
MCP-1	Forward: CATCCAGAGCTTGAGTGTGA<br>Reverse: GTTAGCCTTGCCTTTGTTCA
TSLP	Forward: GGAGATTTGAAAGGGGCTAA<br>Reverse: CATTTCCTGAGTACCGTCAT
GAPDH	Forward: GATGACATCAAGAAGGTGGT<br>Reverse: TACCAGGAAATGAGCTTGAC

Table S2. Active compounds of *S. chinensis*

No.	Molecule Name	PubChem CID	MW	OB (%)	DL
1	(+)-alpha-Longipinene	12311396	204.39	57.47	0.12
2	(+)-Aristolone	12305213	218.37	43.91	0.13
3	(+)-Calarene	15560279	204.39	52.16	0.11

4	(4E,8E,11E,15E)-octadeca-4,8,11,15-tetraenoic acid	5315978	276.46	44.01	0.15
5	3'-Methoxy-4',5,7-trihydroxy-6-(beta-D-glucopyranosyloxy) flavone	5318663	478.44	20.9	0.83
6	6,7,14-Trihydroxy-7,20-epoxykaur-16-en-15-one	433636	348.48	47.72	0.53
7	8-Isopropyl-1,3-dimethyltricyclo(4.4.0.02,7)dec-3-ene	12303902	204.39	29.47	0.12
8	Angeloylgomisin O	91864462	498.62	31.97	0.85
9	Aristolone	165536	218.37	45.31	0.13
10	Besigomsin	3001662	416.51	30.69	0.78
11	beta-Citraurin	9845703	432.7	20.53	0.56
12	beta-Gurjunene	6450812	204.39	51.36	0.1
13	Deoxyharringtonine	285342	515.66	39.27	0.81
14	Deoxyshikonin	98914	272.32	73.85	0.18
15	Dibutyl Phthalate	3026	278.38	64.54	0.13
16	Gomisin G	14992067	508.61	32.68	0.83
17	Gomisin R	11495015	400.46	34.84	0.86
18	Schisandrin C	119112	384.46	46.27	0.84
19	Spathulenol	92231	220.39	82.33	0.12
20	Tigloylgomisin P	5318785	514.62	30.71	0.83
21	Wyerone	643733	258.29	79.24	0.13

MW, molecular weight; OB, oral bioavailability; DL, drug likeness.

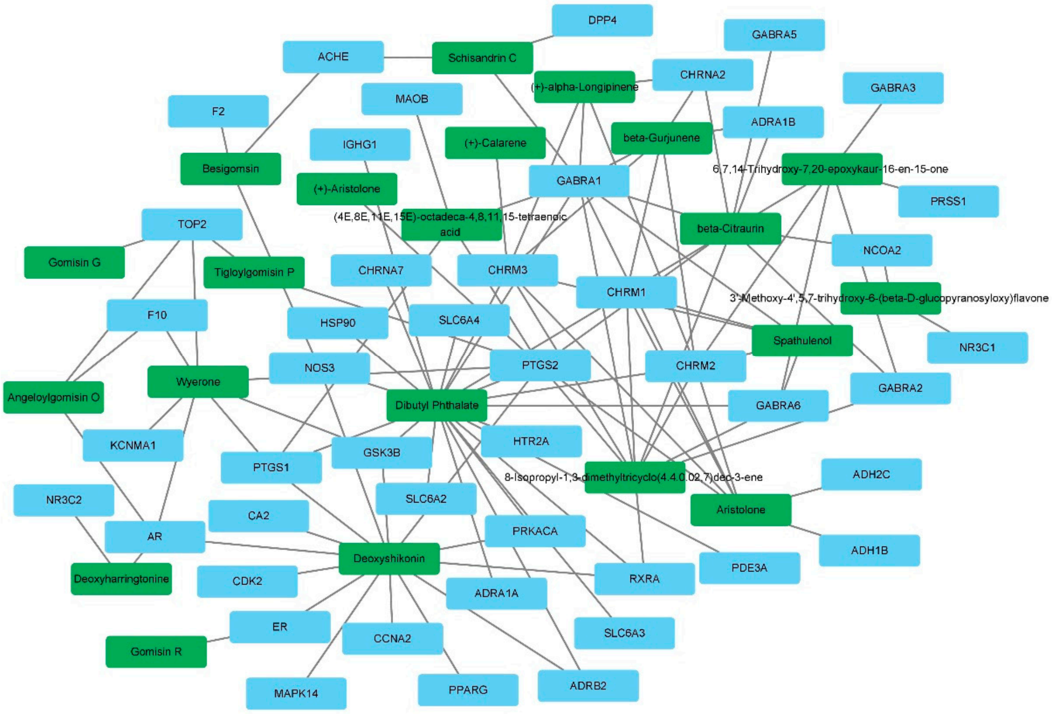

Figure S2. Com-target network

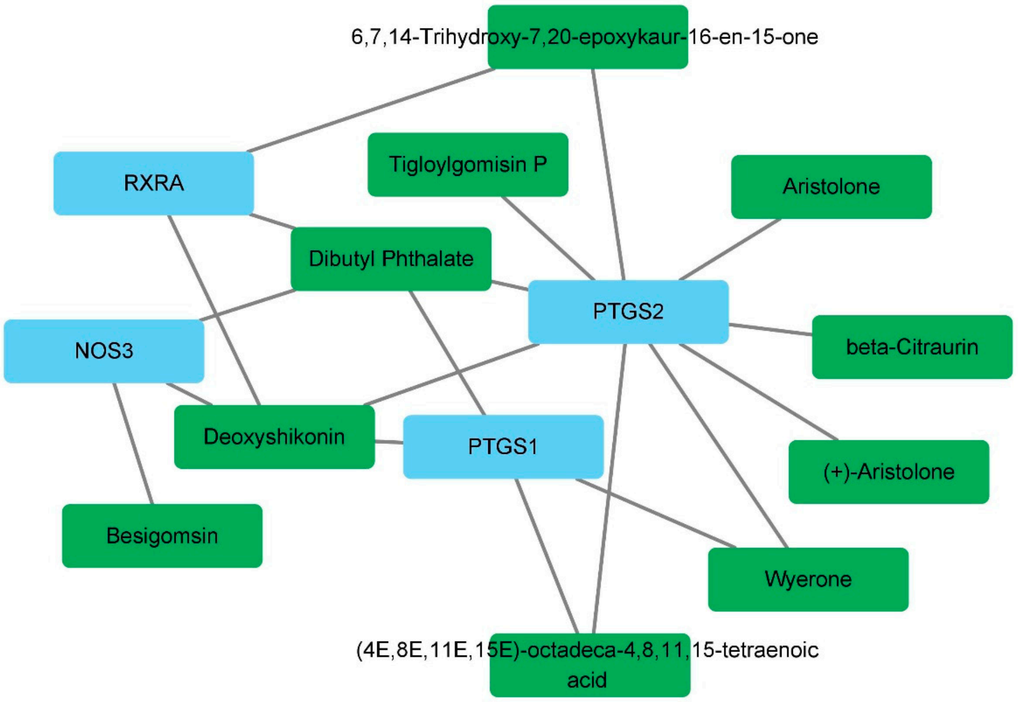

Figure S3. Com-target network (inflammation)
